# Minimally Invasive Tibiotalocalcaneal Arthrodesis with Blocked Retrograde Intramedullary Nail – Report of Three Cases *

**DOI:** 10.1055/s-0041-1731356

**Published:** 2021-10-25

**Authors:** Fernando Delmonte Moreira, Jorge Eduardo de Schoucair Jambeiro, Antero Tavares Cordeiro, José Augusto Oliveira, Felipe Fernandes Leão, Alex Guedes

**Affiliations:** 1 Grupo de Cirurgia do Pé e Tornozelo, Serviço de Ortopedia, Hospital Santa Izabel, Santa Casa de Misericórdia da Bahia, Salvador, BA, Brasil; 2 Programa de Residência Médica em Ortopedia e Traumatologia, Serviço de Ortopedia, Hospital Santa Izabel, Santa Casa de Misericórdia da Bahia, Salvador, BA, Brasil; 3 Grupo de Oncologia Ortopédica, Hospital Santa Izabel, Santa Casa de Misericórdia da Bahia, Salvador, BA, Brasil

**Keywords:** ankle joint, arthrodesis, osteoarthritis, minimally invasive surgical procedures, surgical procedures, operative, ankle

## Abstract

Ankle osteoarthritis (AOA) is associated with pain and variable functional limitation, demanding clinical treatment and possible surgical indication when conservative measures are ineffective – arthrodesis has been the procedure of choice, because it reduces pain, restores joint alignment and makes the segment stable, preserving gait. The present study reports 3 cases (3 ankles) of male patients between 49 and 63 years old, with secondary AOA, preoperative American Orthopaedic Foot and Ankle Society Ankle-Hindfoot Scale (AOFAS AHS) of 27 to 39 points, treated by minimally invasive tibiotalocalcaneal arthrodesis using blocked retrograde intramedullary nail. Hospital stay was of 1 day, and the patients were authorized for immediate loading with removable ambulation orthotics, as tolerated. The physical therapy treatment, introduced since hospitalization, was maintained, prioritizing gait training, strength gain, and proprioception. Clinical and radiographic follow-up was performed at weeks 1, 2, 6, 12 and 24. After evidence of consolidation (between the 6 ^th^ and 10 ^th^ weeks), the orthotics were removed. One patient complained of pain in the immediate postoperative period and, at the end of the 1 ^st^ year, only one patient presented pain during rehabilitation, which was completely resolved with analgesics. Currently, the patients do not present complaints, returning to activities without restrictions – one of them, to the practice of soccer and rappelling. The postoperative AOFAS AHS was from 68 to 86 points.

## Introduction

Primary ankle osteoarthritis (AOA) is rare, and its secondary form 1 is common for traumatic injuries, Charcot, rheumatoid arthritis, and avascular necrosis. 2 3

There are numerous treatment options for AOA, from clinical to surgical management, when conservative measures have no effect – the main options for open treatment include arthrodesis and replacement and distraction arthroplasties. 4

Arthrodesis has been the procedure of choice for reducing pain, restoring alignment and stabilizing the segment, preserving gait.

Ankle arthrodesis can be performed using different types of implants and different access routes, using or not grafts or bone substitutes. 2 3 4

Minimally invasive tibiotalocalcaneal arthrodesis (TTCA) by means of blocked retrograde intramedullary nail (BRIMN) has been indicated due to its biomechanical (shared load, greater bending stiffness, dynamic compression, and rotational stability) and biological (large bone contact area, minimally invasive procedure, articular opening that produces osteocartilaginous "syrup" with hematopoietic potential) advantages. 3 5

The aim of the present study is to report the cases of three patients with secondary AOA (three ankles) submitted to minimally invasive TTCA, using BRIMN.

## Description of Cases

**Fig. 1:**
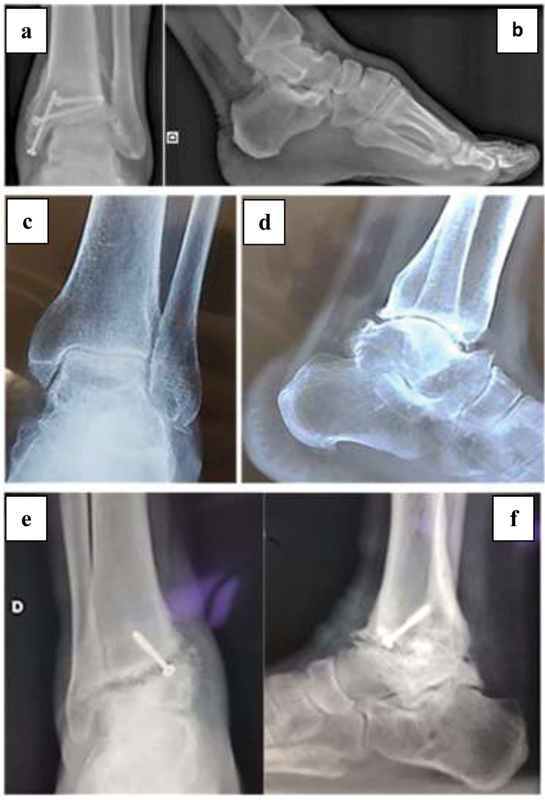
Preoperative radiographic aspect: case 1 – sequela of tibial pylon fracture (a, b); case 2 – chronic ankle instability (c, d); and case 3 – failure in tibiotalar arthrodesis (e, f).

Three patients (three ankles) with secondary AOA, attended at our institution, were treated by minimally invasive TTCA using BRIMN, after failures in conservative measures (cases 1 and 2) and arthrodesis failure (case 3) ( Fig. 1 ), in 2017.

All patients were male, aged between 49 and 63 years old, with variable functional pain and limitation. The American Orthopedic Foot and Ankle Society Ankle-Hindfoot Scale (AOFAS AHS) 6 was between 27 and 39 points ( Table 1 ).

**Table 1:** Description of clinical findings and pre- and postoperative American Orthopaedic Foot and Ankle Society Ankle-Hindfoot Scale and complications

	*Case 1*	*Case 2*	*Patient 3*
***Gender***	Male	Male	Male
***Age (years old)***	49	61	63
***Main complaint***	Pain	Pain	Pain
***Side***	Left	Left	Right
***Arc of motion***	5th	25th	10th
***Deformity***	Valgus	Neutral	Valgus
***Diagnosis***	Sequela of tibial pylon fracture	Chronic ankle instability	Failure in tibiotalar arthrodesis
***Load start (weeks)***	1	1	1
***Consolidation time (weeks)***	10	6	8
***Preoperative AOFAS***	39	33	27
***Postoperative AOFAS***	68	72	86
***Early complication***	No	Pain, resolved with painkillers	No
***Late complication***	No	No	Pain, resolved with painkillers

Abbreviation: AOFAS, American Orthopaedic Foot and Ankle Society Ankle-Hindfoot Scale

**Fig. 2:**
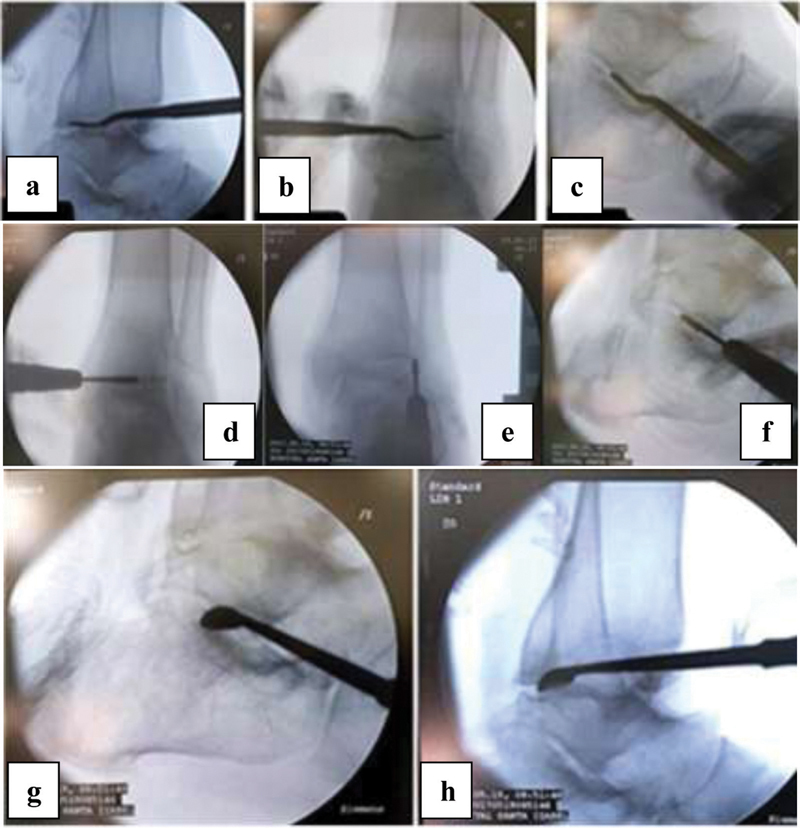
Identification of the tibiotalar and subtalar joints through the respective portals (a, b, c). A 4.3 mm motorized conical cutter was introduced for joint opening (d, e, f). Complementation of opening with curettes, exposing the subcondral bone (g, h).

The patients were positioned in supine position in a radiotransparent surgical table, under sedation, blockade and antibiotic prophylaxis, without ischemia or traction. The joints were accessed by three portals, one subtalar and two tibiotalars (anterolateral and anteromedial), previously marked under fluoroscopy with a disposable 40 × 12mm needle. Incisions of 1.0 to 1.5 cm were made in the markings and dissection blunt to the joint capsules was performed, expanding the work area ( Fig. 2 ). A 4.3 mm motorized conical cutter was introduced for joint opening, complemented with curettes and osteotomes, exposing the subcondral bone. Joint preparation was completed by perforations with a 2.0 mm K-wire in the talar dome and tibial joint surface ( Fig. 2). It was fixed with BRIMN, in the traditional way. Skin sutures and compressive dressing were made. All procedures evolved without issues.

The patients were discharged on the 1 ^st^ postoperative (PO) day. Immediate load initiated, as tolerated, using removable orthotics for ambulation. The stitches were removed on the 15 ^th^ PO day.

Physical therapy treatment was introduced during hospitalization and continued in outpatient care, prioritizing gait training, strength gain, and proprioception.

**Fig. 3:**
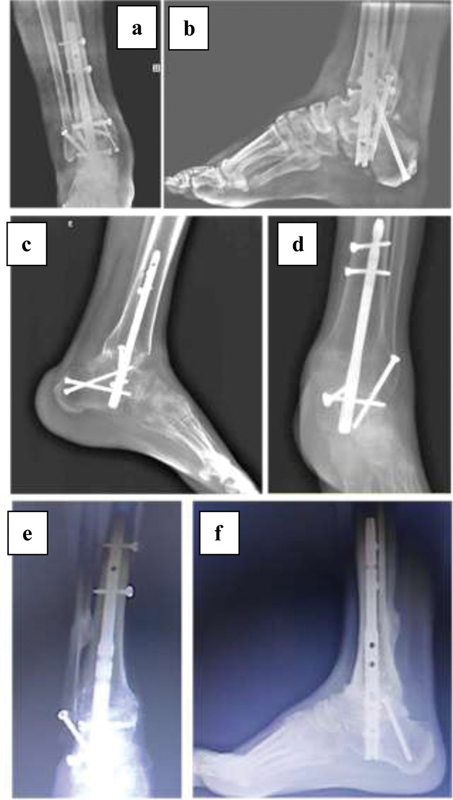
Radiographic aspect in the late postoperative period of cases 1 (a, b), 2 (c, d) and 3 (e, f).

**Fig. 4:**
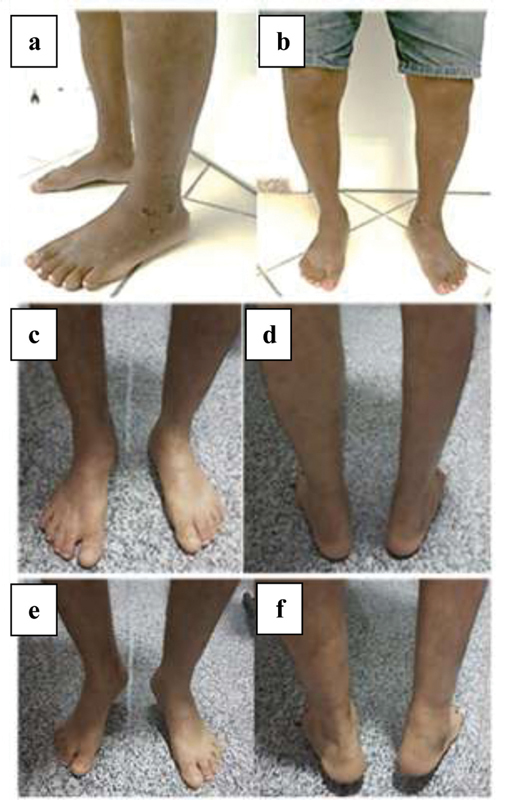
Postoperative aspect of case 2 (a, b) and case 3 (c, d, and, f).

Clinical and radiographic follow-ups ( Fig. 3 ) were performed at weeks 1, 2, 6, 12 and 24. After evidence of consolidation, between the 6 ^th^ and 10 ^th^ weeks, the orthotics were removed ( Fig. 4 ).

One patient complained of pain in the immediate PO, which was resolved with analgesics. At the end of the 1 ^st^ year, only 1 patient presented with pain during rehabilitation, which was completely resolved with analgesics. Currently (3rd PO year), the patients do not present complaints.

All patients returned to activities without restrictions – one of them, to sports (soccer and rappelling). The postoperative AOFAS AHS 6 was between 68 and 86 points ( Table 1 ).

## Discussion

Open TTCA is well-established in the treatment of AOA, regardless of the cause. It is an excellent alternative in the treatment of patients with poor preoperative conditions (low bone stock, misalignment of the hindfoot or history of multiple procedures).

However, despite providing ample exposure, it demands a longer hospital stay and is subject to complications such as infection, dehiscence, and pseudarthrosis, enhanced by comorbidities, often present in patients undergoing this procedure. 3

In a study with 20 patients submitted to open TTCA with BRIMN by AOA, Charcot and deformities, consolidation was observed in 80% of the tibiotalar and subtalar joints and in 20% of the tibiocalcaneans. The AOFAS AHS progressed from 54.20 ± 15.71 to 76.0 ± 11.63 ( *p* < 0.001). The average hospital stay was 6.7 days. There was a high rate of complications, especially infections (35%), culminating in an amputation below the knee. 7

Rammelt et al. 8 evaluated open TTCA with BRIMN in 38 patients, by nonunion, AOA, deformity, Charcot and postarthroplasty failure. They described adequate alignment in 92% and fusion in 84% of the patients. The average stay was 8.4 days. They identified a risk of 24% of at least one postoperative complication, nonunion being the most common, followed by problems with implants and infection.

In a retrospective study, 9 29 patients with deformity underwent open TTCA with BRIMN, obtaining joint consolidation in 96.6%. There was an average increase in the AOFAS AHS from 29.7 to 74.3 ( *p* < 0.01). As complications, three cases of tibial stress, three cases of neuropraxia, and three of infection.

Given the potential for complications, some authors advocate minimally invasive approaches, including percutaneous TTCA through BRIMN. 3 5

Biz et al. 5 presented 28 patients treated with TTCA by percutaneous BRIMN, most of them by post-traumatic AOA. They observed 100% consolidation and 92.85% of plantigrade and stable alignment. As complications, there were one case of screw Protrusion and one case of consolidation retardation, with associated pain.

A systematic review with meta-analysis 2 included 8 patients treated by open TTCA and 15 by the arthroscopic approach. Three patients submitted to open TTCA and four to the arthroscopic approach had plantar ulcers. The fusion rates were similar (75 versus 67%; *p* = 0.679). Complications occurred in 63% of open TTCAs (80% infections) and in 33% of the arthroscopic TTCA (100% nonunion). The presence of ulcers did not influence the genesis of open TTCA infection (67 versus 60%); however, there was a significant increase in nonunion in arthroscopic TTCA (75 versus 18%; *p* = 0.039). Patients without ulcer had a union rate of 80% for both methodologies.

We presented three cases of AOA treated by percutaneous TTCA with BRIMN. The length of stay (1 day) was considerably shorter than that of the literature for an open approach (between 3 and 8 days). The consolidation time (between 6 and 10 weeks) was lower than that of open procedures (12 weeks). The preoperative AOFAS AHS 6 evolved from between 27 and 39 to between 68 and 86, a finding corroborated by the literature for TTCA with BRIMN, 2 3 5 7 8 9 10 only observing early pain (1 patient) and late pain (1 patient), which were resolved after 1 year, without other complications so far.
