# Supplementary material for: Minimally Invasive Tibiotalocalcaneal Arthrodesis with Blocked Retrograde Intramedullary Nail – Report of Three Cases
Source: Rev Bras Ortop (Sao Paulo). 2021 Oct 25;59(1):e143–7. doi: 10.1055/s-0041-1731356 (PMC10957263; doi:10.1055/s-0041-1731356)
Supplement: Related texts

# Research article: Artrodese tibiotalocalcaneana minimamente invasiva com haste intramedular retrógrada bloqueada – Relato de três casos *

**Keywords:** articulação do tornozelo, artrodese, osteoartrite, procedimentos cirúrgicos minimamente invasivos, procedimentos cirúrgicos operatórios, tornozelo

## Abstract

A osteoartrite do tornozelo (OAT) está associada a quadro álgico e limitação funcional variável, demandando tratamento clínico e eventual indicação cirúrgica quando as medidas conservadoras são inefetivas – a artrodese tem sido o procedimento de escolha, por reduzir a dor, restaurar o alinhamento articular e tornar o segmento estável, preservando a marcha. O presente estudo relata 3 casos (3 tornozelos) de pacientes do sexo masculino, com entre 49 e 63 anos de idade, portadores de OAT secundária, American Orthopaedic Foot and Ankle Society Ankle-Hindfoot Scale (AOFAS AHS, na sigla em inglês) pré-operatória de 27 a 39 pontos, tratados mediante artrodese tibiotalocalcaneana minimamente invasiva utilizando haste intramedular retrógrada bloqueada. A permanência hospitalar foi de 1 dia, e os pacientes foram autorizados para carga imediata com órteses removíveis para deambulação, conforme tolerado. O tratamento fisioterápico, introduzido desde o internamento, foi mantido, priorizando-se treino de marcha, ganho de força e propriocepção. Foi realizado acompanhamento clínico e radiográfico nas semanas 1, 2, 6, 12 e 24. Após evidências de consolidação (entre a 6ª e a 10ª semanas), as órteses foram retiradas. Um paciente queixou-se de dor no pós-operatório imediato e, ao final do 1° ano, apenas 1 paciente apresentou dor durante a reabilitação, resolvida completamente com analgésicos. Atualmente, os pacientes não apresentam queixas, retornando às atividades sem restrições – um deles, à prática de futebol e rapel. A AOFAS AHS pós-operatória foi de 68 a 86 pontos.

## Introdução

A osteoartrite do tornozelo (OAT) primária é rara, sendo comum a forma secundária 1 a lesões traumáticas, Charcot, artrite reumatoide e necrose avascular. 2 3

Há diversas opções de tratamento para a OAT, do manejo clínico ao cirúrgico, quando medidas conservadoras não surtem efeito – as principais opções de tratamento cruento incluem a artrodese e as artroplastias de substituição e de distração. 4

A artrodese tem sido o procedimento de escolha, por reduzir a dor, restaurar o alinhamento e estabilizar o segmento, preservando a marcha.

A artrodese do tornozelo pode ser realizada mediante utilização de diversos tipos de implante e diferentes vias de acesso, utilizando ou não enxertos ou substitutos ósseos. 2 3 4

A artrodese tibiotalocalcaneana (ATTC) minimamente invasiva, mediante haste intramedular retrógrada bloqueada (HIMRB) tem sido indicada pelas vantagens biomecânicas (carga compartilhada, maior rigidez à flexão, compressão dinâmica e estabilidade rotacional) e biológicas (grande área de contato ósseo, procedimento minimamente invasivo, cruentização articular que produz “calda” osteocartilaginosa com potencial hematopoiético). 3 5

O objetivo do presente estudo é relatar os casos de três portadores de OAT secundária (três tornozelos) submetidos à ATTC minimamente invasiva, utilizando HIMRB.

## Descrição Dos Casos

Três pacientes (3 tornozelos) portadores de OAT secundária, atendidos em nossa instituição, foram tratados mediante ATTC minimamente invasiva utilizando HIMRB, após falhas nas medidas conservadoras (casos 1 e 2) e falência de artrodese (caso 3) ( Fig. 1 ), no ano de 2017.

**Fig. 1:** Aspecto radiográfico pré-operatório: caso 1 - sequela de fratura do pilão tibial (a, b); caso 2 - instabilidade crônica do tornozelo (c, d); e caso 3 - falha na artrodese tibiotalar (e, f).

Todos eram do sexo masculino, com entre 49 e 63 anos, com dor e limitação funcional variável. A American Orthopaedic Foot and Ankle Society Ankle-Hindfoot Scale (AOFAS AHS, na sigla em inglês) 6 era de 27 a 39 pontos ( Tabela 1 ).

**Tabela 1:** Descrição dos achados clínicos e American Orthopaedic Foot and Ankle Society Ankle-Hindfoot Scale pré e pós-operatórios e complicações

	*Caso 1*	*Caso 2*	*Paciente 3*
***Gênero***	Masculino	Masculino	Masculino
***Idade (Anos)***	49	61	63
***Queixa Principal***	Dor	Dor	Dor
***Lado***	Esquerdo	Esquerdo	Direito
***Arco de Movimento***	5°	25°	10°
***Deformidade***	Valgo	Neutro	Valgo
***Diagnóstico***	Sequela de fratura do pilão tibial	Instabilidade crônica do tornozelo	Falha na artrodese tibiotalar
***Início de Carga (Semanas)***	1	1	1
***Tempo de Consolidação (Semanas)***	10	6	8
***AOFAS Pré-operatório***	39	33	27
***AOFAS Pós-operatório***	68	72	86
***Complicação Precoce***	Não	Dor, resolvida com analgésicos	Não
***Complicação Tardia***	Não	Não	Dor, resolvida com analgésicos

Abreviação: AOFAS, American Orthopaedic Foot and Ankle Society Ankle-Hindfoot Scale

Os pacientes foram posicionados em decúbito dorsal em mesa cirúrgica radiotransparente, sob sedação, bloqueio e antibioticoprofilaxia, sem isquemia ou tração. As articulações foram acessadas mediante três portais, um subtalar e dois tibiotalares (anterolateral e anteromedial), previamente marcados sob fluoroscopia com agulha descartável 40 × 12 mm. Foram feitas incisões de 1,0 a 1,5 cm nas marcações e dissecção romba até as cápsulas articulares, ampliando a área de trabalho ( Fig. 2 ). Foi introduzida uma fresa cônica motorizada de 4,3 mm para cruentização articular, complementada com curetas e osteótomos, expondo o osso subcondral. O preparo articular foi finalizado realizando-se perfurações com fio-K de 2,0 mm no domo talar e na superfície articular tibial ( Fig. 2 ). Foi realizada fixação com HIMRB, da maneira tradicional. Foram feitas suturas de pele e curativo compressivo. Todos os procedimentos evoluíram sem intercorrências.

**Fig. 2:** Identificação das articulações tibiotalar e subtalar através dos respectivos portais (a, b, c). Introduzida fresa cônica motorizada de 4,3 mm para cruentização articular (d, e, f). Complementação da cruentização com curetas, expondo o osso subcondral (g, h).

Alta no 1° dia pós-operatório (PO). Foi iniciada carga imediata, conforme tolerado, utilizando órteses removíveis para deambulação. Os pontos foram retirados no 15° dia PO.

O tratamento fisioterápico foi introduzido no internamento e seguiu ambulatorialmente, priorizando-se o treino de marcha, ganho de força e propriocepção.

Foi feito acompanhamento clínico e radiográfico ( Fig. 3 ) nas semanas 1, 2, 6, 12 e 24. Após evidências de consolidação, entre a 6ª e 10ª semanas, as órteses foram retiradas ( Fig. 4 ).

**Fig. 3:** Aspecto radiográfico no pós-operatório tardio dos casos 1 (a, b), 2 (c, d) e 3 (e, f).

**Fig. 4:** Aspecto pós-operatório do caso 2 (a, b) e caso 3 (c, d, e, f).

Um paciente queixou-se de dor no PO imediato, resolvida com analgésicos. Ao final do 1° ano, apenas 1 paciente apresentou dor na reabilitação, resolvida completamente com analgésicos. Atualmente (3° ano PO) os pacientes não apresentam queixas.

Todos os pacientes voltaram às atividades sem restrições – um deles, à prática desportiva (futebol e rapel). A AOFAS AHS 6 pós-operatória foi de 68 a 86 pontos ( Tabela 1 ).

## Discussão

A ATTC aberta está bem estabelecida no tratamento da OAT, independente da causa, e constitui uma excelente alternativa no tratamento de pacientes com condições pré-operatórias ruins (baixo estoque ósseo, desalinhamento do retropé ou histórico de múltiplos procedimentos).

Entretanto, apesar de prover ampla exposição, a ATTC aberta demanda maior permanência e está sujeita a complicações como infecção, deiscência e pseudartrose, potencializadas por comorbidades, frequentemente presentes nos pacientes submetidos a este procedimento. 3

Em estudo com 20 pacientes submetidos à ATTC aberta com HIMRB por OAT, Charcot e deformidades, observou-se união em 80% das articulações tibiotalares e subtalares e em 20% das tibiocalcaneanas. A AOFAS AHS progrediu de 54.20 ± 15.71 para 76.0 ± 11.63 ( *p* < 0.001). A permanência média foi de 6,7 dias. Houve alto índice de complicações, principalmente infecções (35%), culminando com uma amputação abaixo do joelho. 7

Rammelt et al. 8 avaliaram a ATTC aberta com HIMRB em 38 pacientes, por não união, OAT, deformidade, Charcot e falha pós-artroplastia. Os autores descreveram alinhamento adequado em 92% e fusão em 84% dos pacientes. A permanência média foi de 8,4 dias. Foi identificado um risco de 24% para pelo menos uma complicação pós-operatória, sendo não união a mais comum, seguida por problemas com implantes e infecção.

Em estudo retrospectivo, 9 29 pacientes com deformidade foram submetidos a ATTC aberta com HIMRB, obtendo-se fusão articular em 96,6%. Houve aumento médio da AOFAS AHS de 29,7 para 74,3 (p < 0.01). Como complicações, três casos de *stress* tibial, três de neuropraxia e três de infecção.

Diante do potencial para complicações, alguns autores advogam abordagens minimamente invasivas, dentre elas a ATTC percutânea mediante HIMRB. 3 5

Biz et al. 5 apresentaram 28 pacientes tratados com ATTC mediante HIMRB percutânea, a maioria por OAT pós-traumática, e observaram 100% de consolidação e 92,85% de alinhamento plantígrado e estável. Como complicações, um caso de protrusão de parafuso e um de retardo de consolidação, com dor associada.

Uma revisão sistemática com metanálise 2 incluiu 8 pacientes tratados mediante ATTC aberta e 15 por via artroscópica. Três pacientes submetidos a ATTC aberta e quatro a artroscópica apresentavam úlcera plantar. As taxas de fusão foram similares (75 *versus* 67%; *p* = 0.679). Complicações ocorreram em 63% das ATTC abertas (80% infecções) e em 33% das artroscópicas (100% não união). A presença de úlcera não influenciou na gênese de infecção nas ATTC abertas (67 *versus* 60%); entretanto, houve aumento significativo de não união nas artroscópicas (75 *versus* 18%; *p* = 0.039). Pacientes sem úlcera apresentaram taxa de união de 80% para ambas as metodologias.

Apresentamos três casos de OAT tratados mediante ATTC percutânea com HIMRB. O tempo de permanência (1 dia) foi consideravelmente menor do que o da literatura para a abordagem aberta (3 a 8 dias). O tempo de consolidação (6 a 10 semanas) foi inferior aos procedimentos cruentos (12 semanas). A AOFAS AHS 6 pré-operatória de 27 a 39 evoluiu para 68 a 86, achado corroborado pela literatura para a ATTC com HIMRB, 2 3 5 7 8 9 10 sendo observada apenas dor precoce (1 paciente) e dor tardia (1 paciente), após 1 ano, as quais foram resolvidas, sem outras complicações até o momento.
